# Facile Synthesis of Carbon Cloth Supported Cobalt Carbonate Hydroxide Hydrate Nanoarrays for Highly Efficient Oxygen Evolution Reaction

**DOI:** 10.3389/fchem.2021.754357

**Published:** 2021-08-27

**Authors:** Yubing Yan

**Affiliations:** Department of Chemistry and Chemical Engineering, Luliang University, Lvliang, China

**Keywords:** cobalt carbonate hydroxide hydrate, needle-like nanoarrays, porous structure, electrocatalyst, oxygen evolution reaction

## Abstract

Developing efficient and low-cost replacements for noble metals as electrocatalysts for the oxygen evolution reaction (OER) remain a great challenge. Herein, we report a needle-like cobalt carbonate hydroxide hydrate (Co(CO_3_)_0.5_OH·0.11H_2_O) nanoarrays, which *in situ* grown on the surface of carbon cloth through a facile one-step hydrothermal method. Scanning electron microscopy (SEM) and transmission electron microscopy (TEM) characterizations demonstrate that the Co(CO_3_)_0.5_OH nanoarrays with high porosity is composed of numerous one-dimensional (1D) nanoneedles. Owing to unique needle-like array structure and abundant exposed active sites, the Co(CO_3_)_0.5_OH@CC only requires 317 mV of overpotential to reach a current density of 10 mA cm^−2^, which is much lower than those of Co(OH)_2_@CC (378 mV), CoCO_3_@CC (465 mV) and RuO_2_@CC (380 mV). For the stability, there is no significant attenuation of current density after continuous operation 27 h. This work paves a facile way to the design and construction of electrocatalysts for the OER.

## Introduction

As a key anodic reaction, oxygen evolution reaction (OER) plays an important role in energy-relative electrochemical conversion technologies, such as water splitting and rechargeable Zn–air batteries (Song et al., 2020; Wu et al., 2020). However, OER suffers from sluggish kinetics owing to four electron transfer process, which significantly increases the overpotential. (Fu et al., 2018; Xiao et al., 2019; Liu et al., 2020). To facilitate the OER, the noble metal-based materials like IrO_2_ and RuO_2_ have been regarded as highly active catalysts for the OER. (Xie et al., 2019; Du et al., 2021; Wang et al., 2021). Although they present outstanding activity for the OER, the high cost, scarcity reserves and poor stability are main reasons to limit their practical applications. Therefore, it is highly urgent to explore and develop the cost-effective and earth-abundant electrocatalysts for the OER.

For the past few years, cobalt-based transition metal materials, such as cobalt oxides (CoO_x_) (Xu et al., 2016; Li et al., 2018), cobalt phosphides (CoP_x_) (Chen et al., 2019; Jin et al., 2019), cobalt sulfide (CoS_x_) (Wang et al., 2015; Zhang H. et al., 2020), cobalt nitride (CoN_x_) (Chen et al., 2016; Liu et al., 2021), and cobalt hydroxides (Co(OH)_2_) Dileep et al. (2020), Qin et al. (2020) have been widely investigated as non-noble metal electrocatalysts for the OER. More recently, cobalt carbonate hydroxide hydrate [Co(CO_3_)_0.5_OH·0.11H_2_O] have received far-ranging attention as the OER catalysts (Zhang et al., 2015; Wang et al., 2017; Zhang S. et al., 2020), and not rather than only as a precursor to produce oxides and phosphides. However, the low conductivity and deficient catalytically active sites of Co(CO_3_)_0.5_OH·0.11H_2_O limit its intrinsic OER activity in energy-relative electrochemical devices. To address such problems, the introducing carbon support and morphology modulation should be two important strategies for improving the OER activity of transition metal-based materials. Through the introducing carbon support, the electronic contact between the carbon support and the active materials can induce the charge redistribution and the changes in electronic structure of the active materials, probably leading to improve the electronic conductivity and facilitate charge transfer during the OER (Liu et al., 2018; Yang et al., 2020). For the morphology modulation, three-dimensional (3D) porous array structure presents significant advantages among various morphology, including large specific surface area, abundant exposed catalytically active sites, excellent structure stability, which are highly favorable for the mass and charge transfer during electrocatalytic reactions (Xu et al., 2018; Wang et al., 2020). Besides, 1D-nano structure have inherent structural advantages, such as high specific surface area, fast electron and material transport, low solubility and difficult agglomeration, etc. Therefore, 1D-nanostructures are widely used in electrocatalytic applications. However, it still exists a big challenge to realize deliberate control over the above two features in a facile and efficient method.

Herein, we report the anchoring of needle-like Co(CO_3_)_0.5_OH·0.11H_2_O nanoarrays on carbon cloth [Co(CO_3_)_0.5_OH@CC] with the assistance of urea and NH_4_F through a facile one-step hydrothermal method. Urea and NH_4_F serve as both effective agents to help to favor the formation of well-defined needle-like array structure. The geometric and electronic structure are explored in detail by different characterization methods. Benefitted from needle-like array structure and abundant exposed active sites, the developed Co(CO_3_)_0.5_OH@CC displays superior electrocatalytic performance towards OER with a low overpotential of 317 mV at 10 mA cm^−2^ and a long-term stability.

## Experimental Section

### Reagents and Chemicals

All reagents and chemicals in the experimental sections were used without further purification. Cobalt nitrate hexahydrate [Co(NO_3_)_2_⋅6H_2_O] was purchased from Macklin Biochemical Co., Ltd. (Shanghai, China). Urea [CO(NH_2_)_2_] was brought from Beijing Solarbio Science and Technology Co., Ltd. (Beijing, China). Ammonium fluoride (NH_4_F) and ethanol were obtained from Sinopharm Chemical Reagent Co., Ltd. (Shanghai, China). Cobalt (Ⅱ) carbonate hydrate (CoCO_3_⋅H_2_O), Cobalt (Ⅱ) hydroxide (Co(OH)_2_), commercial ruthenium (IV) oxide (RuO_2_) were purchased from Aladdin Ltd. (Shanghai, China).

### Synthesis of the Co(CO_3_)_0.5_OH@CC

The Co(CO_3_)_0.5_OH@CC was fabricated by a facile one-step hydrothermal method. In a typical preparation procedure, 1.5 mmol Co(NO_3_)_2_⋅6H_2_O was dissolved in 30 ml deionized water by magnetic stirring for several minutes to form a homogeneous solution. Then, 0.1 g NH_4_F and 0.3 g urea were added to the reactor under vigorous string for 5 min. The obtained clear solution was transferred into a Teflon-lined stainless-steel autoclave and a piece of 2*4 cm^2^ carbon cloth was immersed into the solution vertically. The autoclave was then sealed and placed in an oven at 120 ^o^C for 8 h. After cooled to room temperature, the as-fabricated Co(CO_3_)_0.5_OH@CC was taken out and rinsed with ethanol and deionized water for several times, and dried at 40 ^o^C overnight.

### Characterization

X-ray diffraction (XRD) measurement was performed on X-ray powder diffractometer with a Cu K_ɑ_ radiation (λ = 1.5406 Å). Scanning electron microscopy (SEM) images were collected on Hitachi S5500 scanning electron microscope. Transmission electron microscope (TEM) and high-resolution transmission electron microscope (HRTEM) images were collected using a JEOL JEM2100F (accelerating voltage of 200 kV). Element dispersive spectroscopy (EDS) measurements and line scans profiles were performed on FEI Tecnai G2 F20 microscope, an accessory built on the JEOL JEM-2100F. All XPS analyses were carried out with Thermo VG Scientific ESCALAB 250 spectrometer (Al Kα radiator).

### Electrochemical Measurement

Electrochemical properties of all catalysts were studied with a standard three-electrode system on CHI 760E electrochemical analyzer (Shanghai Chenghua Co.). A saturated calomel electrode (SCE) and a graphite rod were employed as the reference electrode and the auxiliary electrode, respectively. All the potentials involved in this manuscript were converted to the reversible hydrogen electrode (RHE) scale by following equation: E_RHE_ = E_SCE_ + 0.0592*pH + 0.242. All the potentials involved in this manuscript were not corrected by *iR*-correction. Before the test, 1.0 M KOH was saturated by high-purity O_2_. A catalysts-modified carbon cloth (1*1 cm^2^) was used as working electrode. Linear sweep voltammetry (LSV) measurement was performed with a sweep rate of 5 mV s^−1^. The *C*_dl_ value of different catalysts were performed at potential of 1.02 to 1.12 V with the cyclic voltammograms at different sweeping rates from 2 mV s^−1^–10 mV s^−1^. The electrochemical impedance spectroscopy (EIS) was collected in a frequency range from 0.01 Hz to 100 kHz at 1.7 V.

## Results and Discussion

**FIGURE 1:**
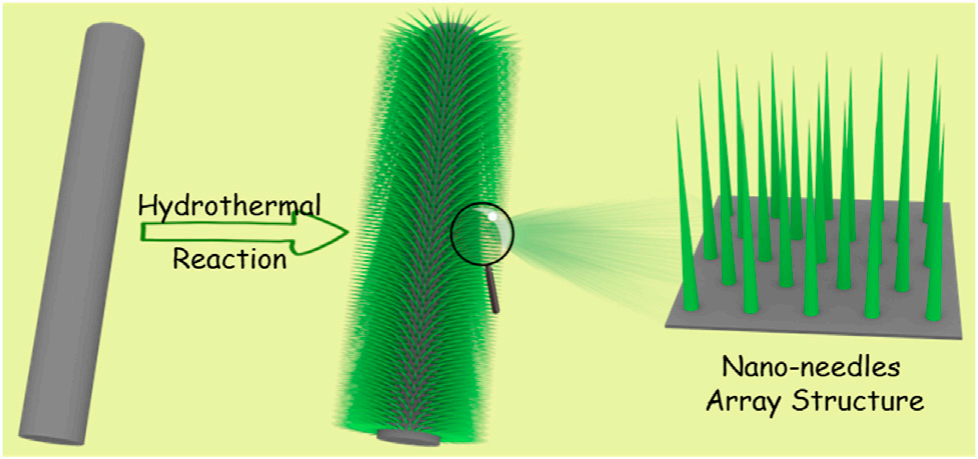
Schematic illustration for the synthesis of Co(CO_3_)_0.5_OH@CC nanoarrays.

The synthesis route of Co(CO_3_)_0.5_OH@CC nanoarrays is illustrated schematically in Figure 1. The needle-like Co(CO_3_)_0.5_OH nanoarrays were *in situ* grown on the surface of carbon cloth through a facile one-step hydrothermal method, where the Co(NO_3_)_2_ aqueous solution were used as precursors and urea as an alkaline reagent in the presence of NH_4_F at 120°C. Urea, a common ammonia-releasing agent, provides hydroxyl ions (OH^−^) and carbonate ions (CO_3_^2−^) during the hydrolysis (Wang et al., 2012). NH_4_F is a good complexing ligand for Co^2+^ that can serve to reduce the concentration of free Co^2+^ ions, lowing the supersaturation to probably favor the gradual growth of needle-like Co(CO_3_)_0.5_OH nanoarrays (Zhu et al., 2013).

**FIGURE 2:**
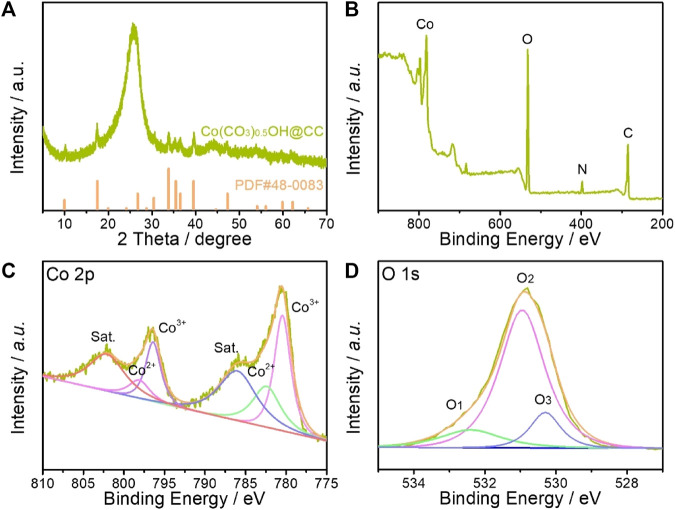
**(A)** XRD pattern of Co(CO_3_)_0.5_OH@CC nanoarrays; **(B)** Full XPS spectrum of Co(CO_3_)_0.5_OH @CC nanoarrays; High-resolution **(C)** Co 2p and **(D)** O 1s XPS spectra.

Figure 2A shows the X-ray powder diffraction (XRD) pattern of as-prepared product, which was utilized to investigate the phase and purity of the as-synthesized Co(CO_3_)_0.5_OH@CC catalysts. Except for the peak about 26° derived from carbon cloth, other identified peaks can be matched well with pure orthorhombic Co(CO_3_)_0.5_OH·0.11H_2_O (JCPDS No. 48–0,083, a = 8.792 Å, b = 10.150 Å and c = 4.433 Å). The surface composition and valence states of Co(CO_3_)_0.5_OH@CC nanoarrays were determined by X-ray photoelectron spectroscopy (XPS). Full XPS spectrum demonstrates the co-existence of Co, O, N and C in Co(CO_3_)_0.5_OH@CC nanoarrays (Figure 2B). Figure 2C shows the high-resolution Co 2p XPS spectrum, which can be best fitted with two spin-orbit doublets and two shakeup satellites. The fitting peaks at 782.40 and 798.10 eV are assigned to Co^2+^ species, whereas the fitting peaks at 780.46 and 796.40 eV are attributed to Co^3+^ species (Li et al., 2020a; Li et al., 2020b). For O 1s XPS spectrum, the peaks located at 530.3, 530.95 and 532.40 eV are associated with metal-oxygen bond, hydroxyl group (OH^−^) and oxygen vacancies (Tang et al., 2020; Li M. et al., 2021). In the as-prepared Co(CO_3_)_0.5_(OH)@CC catalysts, the metal-oxygen bond and hydroxyl group are typical characteristics of carbonate hydroxide hydrates. While the high content oxygen vacancies could also be found in this catalyst, which could offer more efficient active sites and act as oxygen buffer to accelerate the OER kinetics during electrocatalysis process.

**FIGURE 3:**
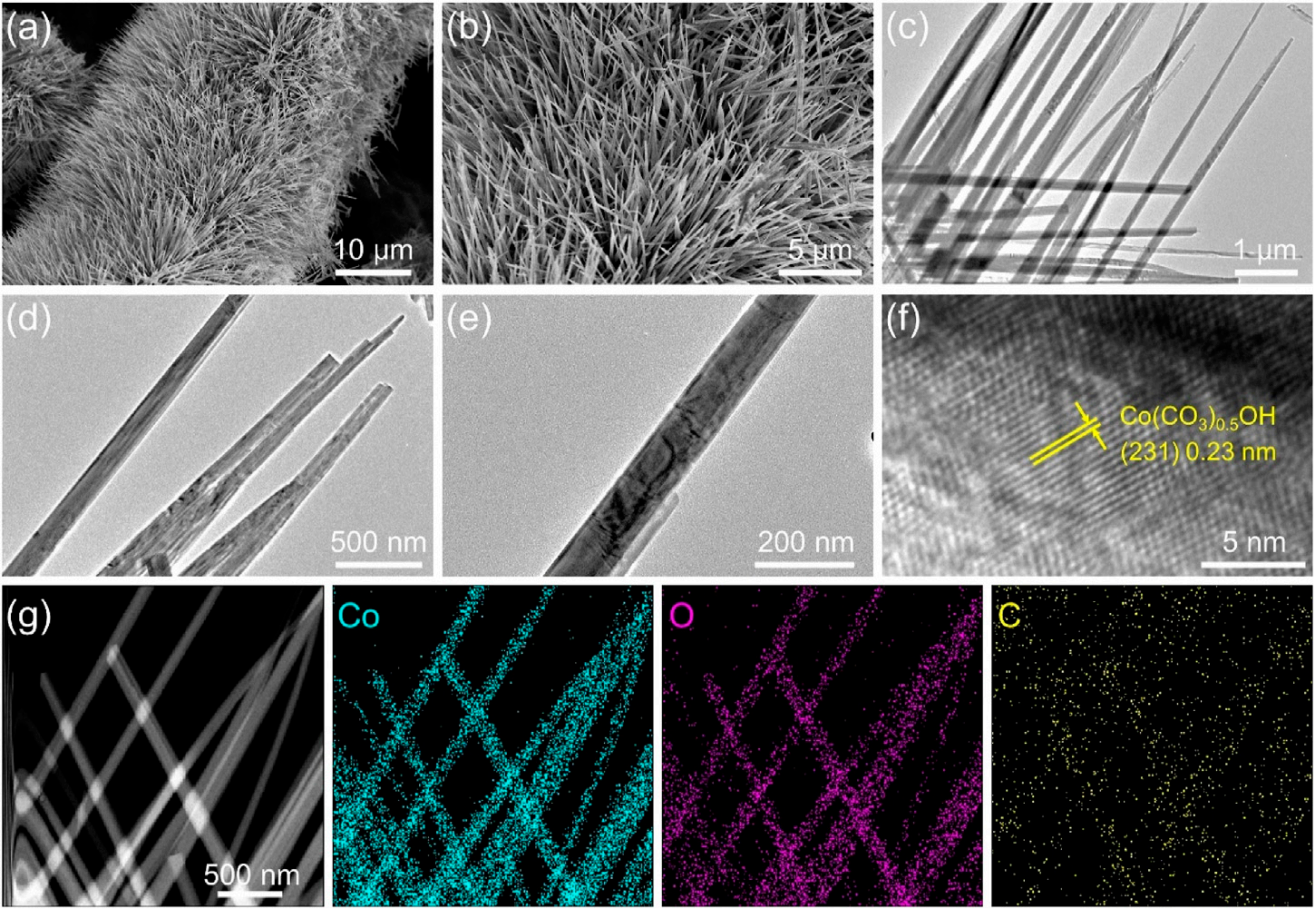
**(A**,**B)** SEM images of Co(CO_3_)_0.5_OH@CC nanoarrays at different magnifications; **(C–E)** TEM images of Co(CO_3_)_0.5_OH nanoneedles at different magnifications; **(F)** HRTEM image of Co(CO_3_)_0.5_OH nanoneedle; **(G)** STEM image and EDX element mappings of Co(CO_3_)_0.5_OH nanoneedles.

The morphology and structure of the product were further characterized with scanning electron microscopy (SEM) and transmission electron microscopy (TEM). From typical SEM images (Figures 3A,B; Supplementary Figure S1), one can see that the as-prepared Co(CO_3_)_0.5_OH·0.11H_2_O exhibits a needle-like array structure and grew vertically and densely on the surface of carbon cloth, forming a 3D network structure. The needle-like structure of Co(CO_3_)_0.5_OH·0.11H_2_O was clearly observed in TEM images shown in Figures 3D,E. The average diameter of Co(CO_3_)_0.5_OH·0.11H_2_O of nanoneedle is about 80 nm. 3D porous array structure can facilitate the mass diffusion and the accessibility of the active sites/electrolytes. Figure 3F shows the high-resolution TEM (HRTEM) image of Co(CO_3_)_0.5_OH·0.11H_2_O of nanoneedle, which presents well-resolved lattice fringes with lattice spacing of about 0.23 nm corresponding to the (231) plane of Co(CO_3_)_0.5_OH·0.11H_2_O. Energy dispersive X-ray (XPS) spectrum confirms the presence of Co and O elements (Supplementary Figure S2), in accordance with XPS results. The element distribution was further investigated by EDX element mappings (Figure 3G; Supplementary Figure S3) and EDX line scanning profiles (Supplementary Figure S4). It can be seen that the Co, O and C elements are homogeneously distributed throughout the Co(CO_3_)_0.5_OH·0.11H_2_O nanoneedles. All the characterizations above prove that Co(CO_3_)_0.5_OH@CC catalyst was successfully synthesized.

**FIGURE 4:**
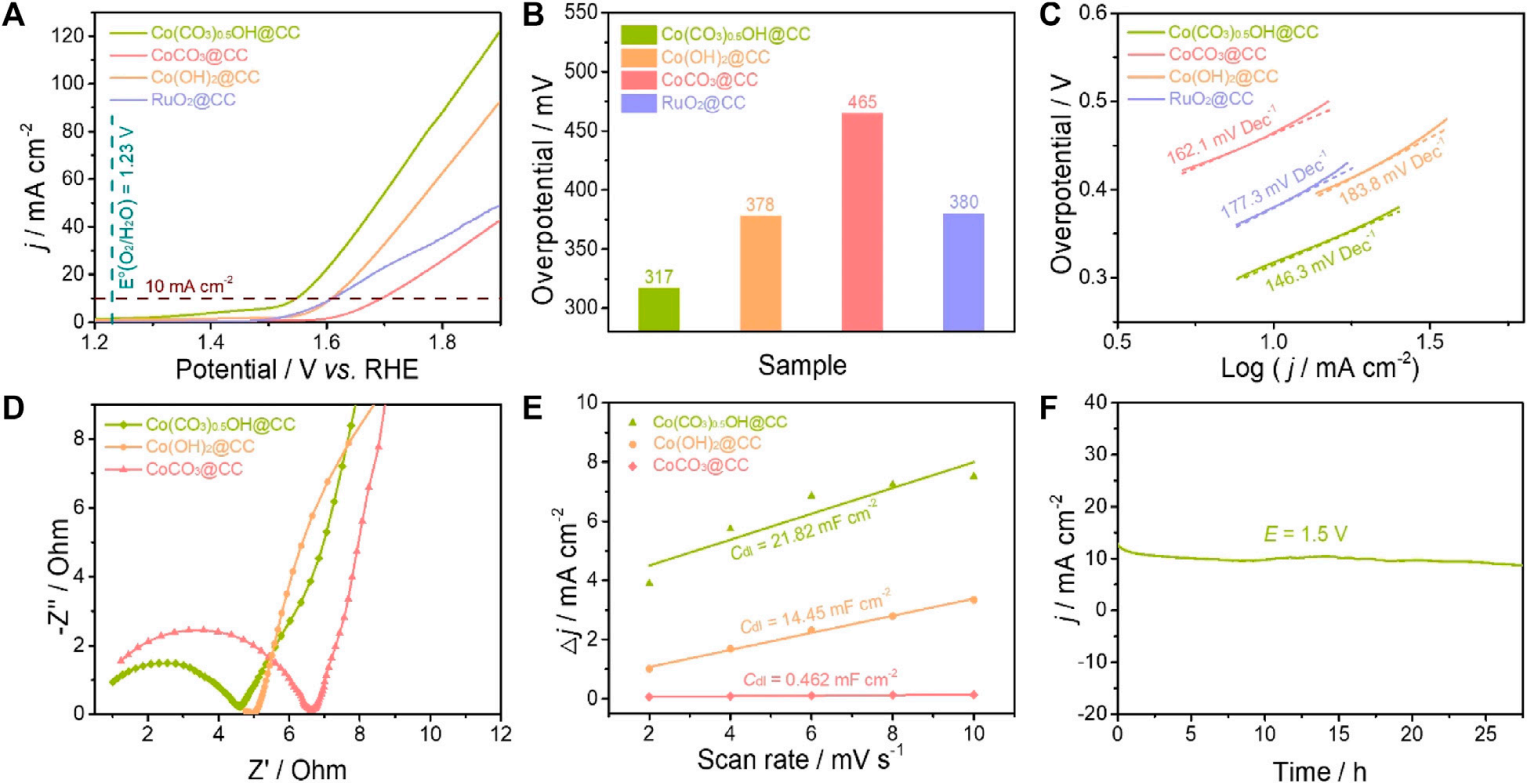
Comparison of the OER activity of catalysts: **(A)** OER polarization curves in O_2_-saturated 1.0 M KOH; **(B)** Overpotentials at 10 mA cm^−2^; **(C)** Tafel plots; **(D)** EIS Nyquist plots; **(E)** *C*_dl_ values of catalysts; **(F)** The chronopotentiometry curve of Co(CO_3_)_0.5_OH@CC obtained at 1.5 V.

The electrocatalytic performance of Co(CO_3_)_0.5_OH@CC nanoarrays for the OER was evaluated using a standard three-electrode configuration in an alkaline medium (1.0 M KOH). For comparison, Co(OH)_2_@CC, CoCO_3_@CC and RuO_2_@CC were also tested under the same condition. Figure 4A displays the OER polarization curves of Co(CO_3_)_0.5_OH@CC, Co(OH)_2_@CC, CoCO_3_@CC and RuO_2_@CC. It can be found that Co(CO_3_)_0.5_OH@CC presents the best electrocatalytic activity towards OER as compared with the rest of samples. The Co(CO_3_)_0.5_OH@CC electrode only require 317 mV of overpotential to reach a current density of 10 mA cm^−2^ (Figure 4B), which is much lower than those of Co(OH)_2_@CC (378 mV), CoCO_3_@CC (465 mV) and RuO_2_@CC (380 mV). The OER reaction kinetics of catalysts was evaluated from the corresponding Tafel plots. As indicated in Figure 4C, the Co(CO_3_)_0.5_OH@CC exhibits the smallest Tafel slope of 146.3 mV dec^−1^, much lower than those of Co(OH)_2_@CC (162.1 mV dec^−1^), CoCO_3_@CC (183.8 mV dec^−1^) and RuO_2_@CC (177.3 mV dec^−1^), demonstrating a more favorable reaction kinetics of Co(CO_3_)_0.5_OH@CC during the OER. The excellent reaction kinetics of Co(CO_3_)_0.5_OH@CC may be associated with good charge transfer rate (Sun et al., 2018). To confirm this point, the electrochemical impedance spectroscopy (EIS) was performed. The Nyquist plot of Co(CO_3_)_0.5_OH@CC presents the smaller semicircles compared to those of Co(OH)_2_@CC and CoCO_3_@CC (Figure 4D), demonstrating the lower charge-transfer resistance (*R*_ct_) and rapider charge transfer rate. The electrochemical double layer capacitance (*C*_dl_) was applied to appraise the magnitude of electrochemical active surface area (ECSA) (Li Z. et al., 2021). The measurements were performed at potential of 1.02–1.12 V with the cyclic voltammograms at different sweeping rates from 2 mV s^−1^ to 10 mV s^−1^ (Supplementary Figure S5). As revealed in Figure 4E, the Co(CO_3_)_0.5_OH@CC has a larger *C*_dl_ value (21.82 mF cm^−2^) than those of Co(OH)_2_@CC (14.45 mF cm^−2^) and CoCO_3_@CC (0.462 mF cm^−2^), suggesting the presence of higher active surface area on Co(CO_3_)_0.5_OH@CC. The long-term stability is another vital parameter for the practical application of electrocatalysts, which was studied via the chronoamperometric *i-t* test conducted at 1.5 V. After continuous operation 27 h, there is no significant attenuation of current density for the Co(CO_3_)_0.5_OH@CC, indicating good electrocatalytic stability.

**FIGURE 5:**
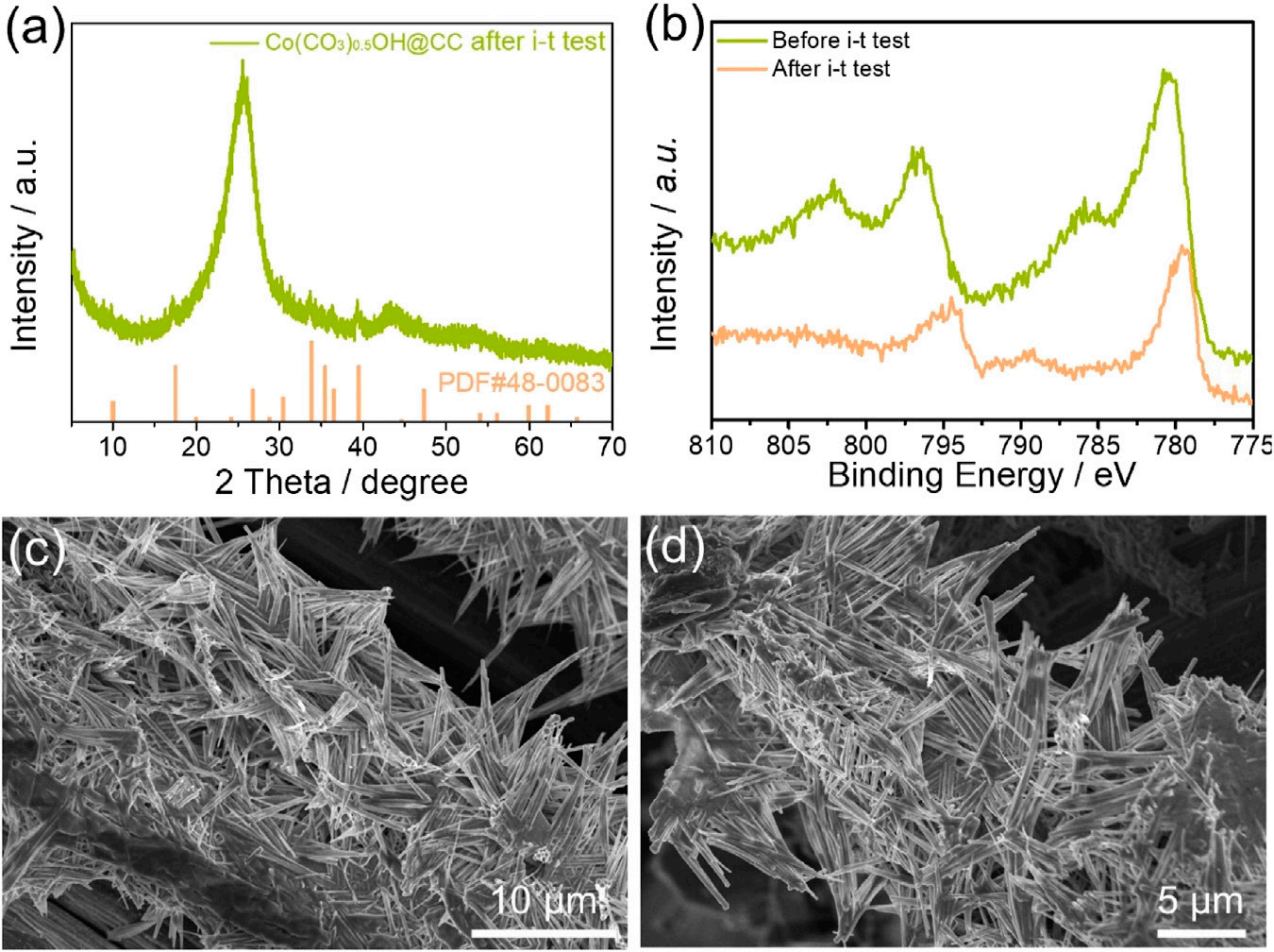
**(A)** XRD pattern of recovered Co(CO_3_)_0.5_OH@CC after *i-t* test; **(B)** High-resolution Co 2p spectra of Co(CO_3_)_0.5_OH@CC before and after *i-t* test; **(C–D)** SEM images of recovered Co(CO_3_)_0.5_OH@CC.

The electrocatalytic stability of Co(CO_3_)_0.5_OH@CC is also evidenced by XRD, XPS and SEM characterizations before and after continuous OER operation. From XRD pattern shown in Figure 5A, the diffraction peak of recovered Co(CO_3_)_0.5_OH@CC is the same as that before the OER stability. Figure 5B shows the high-resolution Co 2p XPS spectra of Co(CO_3_)_0.5_OH@CC before and after test. Compared with that before the reaction, the peaks of Co 2p spectrum for Co(CO_3_)_0.5_OH@CC negatively shift, which suggested that Co^2+^ had a tendency to transform like Co^3+^ during the OER process. The Co^3+^ species may be assigned to the Co species in CoOOH, suggesting CoOOH may serve as active site for the OER (Zhong et al., 2021). Furthermore, the morphology of Co(CO_3_)_0.5_OH@CC is maintained well apart from the slight surface corrosion (Figures 5C,D). According to the above results and data analysis, the Co(CO_3_)_0.5_OH@CC demonstrates outstanding OER activity, which can be its unique needle-like array architecture and surface chemical properties.

## Conclusion

In summary, we reported a novel catalyst consisting of long-needle like carbonate hydroxide hydrate nanoarrays dirrectly *in situ* growth on carbon cloth substrate by a green and facial one-step hydrothermal strategy. Benefiting from the nanoneedles arrayed architecture and unique active component, the as-prepared Co(CO_3_)_0.5_OH@CC possess abundant accessible active sites, efficient mass/electron transfer channels and robust structure stabilities. Thus, the as-synthesized Co(CO_3_)_0.5_OH@CC exhibits outstanding electrocatalytic performance towards the OER in alkaline medium (1.0 M KOH) with a low overpotential of 317 mV at a current density of 10 mA cm^−2^ and could maintained well even after 27 h continuous electrolysis. We believe that such excellent catalytic activity and robust stability of Co(CO_3_)_0.5_OH@CC enables it to be an economical and competent electrocatalyst for large-scale electrochemical applications.

## Data Availability

The original contributions presented in the study are included in the article/Supplementary Material, further inquiries can be directed to the corresponding author.

## References

[B1] Chen J. Fan C. Hu X. Wang C. Huang Z. Fu G. (2019). Hierarchically Porous Co/Co_X_ M_Y_ (M = P, N) as an Efficient Mott-Schottky Electrocatalyst for Oxygen Evolution in Rechargeable Zn-Air Batteries. Small 15, 1901518. 10.1002/smll.201901518 31140732

[B2] Chen P. Xu K. Tong Y. Li X. Tao S. Fang Z. (2016). Cobalt Nitrides as a Class of Metallic Electrocatalysts for the Oxygen Evolution Reaction. Inorg. Chem. Front. 3, 236–242. 10.1039/c5qi00197h

[B3] Dileep N. P. Vineesh T. V. Sarma P. V. Chalil M. V. Prasad C. S. Shaijumon M. M. (2020). Electrochemically Exfoliated β-Co(OH)_2_ Nanostructures for Enhanced Oxygen Evolution Electrocatalysis. ACS Appl. Energ. Mater. 3, 1461–1467. 10.1021/acsaem.9b01901

[B4] Du J. Li F. Sun L. (2021). Metal-organic Frameworks and Their Derivatives as Electrocatalysts for the Oxygen Evolution Reaction. Chem. Soc. Rev. 50, 2663–2695. 10.1039/d0cs01191f 33400745

[B5] Fu G. Tang Y. Lee J.-M. (2018). Recent Advances in Carbon-Based Bifunctional Oxygen Electrocatalysts for Zn−Air Batteries. ChemElectroChem 5, 1424–1434. 10.1002/celc.201800373

[B6] Jin W. Chen J. Liu B. Hu J. Wu Z. Cai W. (2019). Oxygen Vacancy-Rich In‐Doped CoO/CoP Heterostructure as an Effective Air Cathode for Rechargeable Zn-Air Batteries. Small 15, 1904210. 10.1002/smll.201904210 31559688

[B7] Li M. Li H. Jiang X. Jiang M. Zhan X. Fu G. (2021a). Gd-induced Electronic Structure Engineering of a NiFe-Layered Double Hydroxide for Efficient Oxygen Evolution. J. Mater. Chem. A. 9, 2999–3006. 10.1039/d0ta10740a

[B8] Li M. Pan X. Jiang M. Zhang Y. Tang Y. Fu G. (2020a). Interface Engineering of Oxygen-Vacancy-Rich CoP/CeO_2_ Heterostructure Boosts Oxygen Evolution Reaction. Chem. Eng. J. 395, 125160. 10.1016/j.cej.2020.125160

[B9] Li M. Wang Y. Zheng Y. Fu G. Sun D. Li Y. (2020b). Gadolinium‐Induced Valence Structure Engineering for Enhanced Oxygen Electrocatalysis. Adv. Energ. Mater. 10, 1903833. 10.1002/aenm.201903833

[B10] Li Y. Li F.-M. Meng X.-Y. Li S.-N. Zeng J.-H. Chen Y. (2018). Ultrathin Co_3_O_4_ Nanomeshes for the Oxygen Evolution Reaction. ACS Catal. 8, 1913–1920. 10.1021/acscatal.7b03949

[B11] Li Z. Li H. Li M. Hu J. Liu Y. Sun D. (2021b). Iminodiacetonitrile Induce-Synthesis of Two-Dimensional PdNi/Ni@carbon Nanosheets with Uniform Dispersion and strong Interface Bonding as an Effective Bifunctional Eletrocatalyst in Air-Cathode. Energ. Storage Mater. 42, 118–128. 10.1016/j.ensm.2021.07.027

[B12] Liu H. Lei J. Yang S. Qin F. Cui L. Kong Y. (2021). Boosting the Oxygen Evolution Activity over Cobalt Nitride Nanosheets through Optimizing the Electronic Configuration. Appl. Catal. B: Environ. 286, 119894. 10.1016/j.apcatb.2021.119894

[B13] Liu Y. Guan X. Huang B. Wei Q. Xie Z. (2020). One-Step Synthesis of N, P-Codoped Carbon Nanosheets Encapsulated CoP Particles for Highly Efficient Oxygen Evolution Reaction. Front. Chem. 7, 805. 10.3389/fchem.2019.00805 31998679 PMC6962193

[B14] Liu Z. Yu X. Yu H. Xue H. Feng L. (2018). Nanostructured FeNi_3_ Incorporated with Carbon Doped with Multiple Nonmetal Elements for the Oxygen Evolution Reaction. ChemSusChem 11, 2703–2709. 10.1002/cssc.201801250 29892992

[B15] Qin J.-F. Xie J.-Y. Wang N. Dong B. Chen T.-S. Lin Z.-Y. (2020). Surface Construction of Loose Co(OH)_2_ Shell Derived from ZIF-67 Nanocube for Efficient Oxygen Evolution. J. Colloid Interf. Sci. 562, 279–286. 10.1016/j.jcis.2019.12.033 31841887

[B16] Song J. Wei C. Huang Z.-F. Liu C. Zeng L. Wang X. (2020). A Review on Fundamentals for Designing Oxygen Evolution Electrocatalysts. Chem. Soc. Rev. 49, 2196–2214. 10.1039/c9cs00607a 32133479

[B17] Sun Y. Xu K. Wei Z. Li H. Zhang T. Li X. (2018). Strong Electronic Interaction in Dual‐Cation‐Incorporated NiSe 2 Nanosheets with Lattice Distortion for Highly Efficient Overall Water Splitting. Adv. Mater. 30, 1802121. 10.1002/adma.201802121 30129696

[B18] Tang Y. Liu Q. Dong L. Wu H. B. Yu X.-Y. (2020). Activating the Hydrogen Evolution and Overall Water Splitting Performance of NiFe LDH by Cation Doping and Plasma Reduction. Appl. Catal. B: Environ. 266, 118627. 10.1016/j.apcatb.2020.118627

[B19] Wang B. Zhu T. Wu H. B. Xu R. Chen J. S. Lou X. W. (2012). Porous Co3O4 Nanowires Derived from Long Co(CO_3_)_0.5_(OH)·0.11H_2_O Nanowires with Improved Supercapacitive Properties. Nanoscale 4, 2145–2149. 10.1039/c2nr11897a 22337265

[B20] Wang H. Li Z. Li G. Peng F. Yu H. (2015). Co_3_S_4_/NCNTs: A Catalyst for Oxygen Evolution Reaction. Catal. Today 245, 74–78. 10.1016/j.cattod.2014.06.006

[B21] Wang S. Wang Y. Zang S. Q. Lou X. W. (2020). Hierarchical Hollow Heterostructures for Photocatalytic CO_2_ Reduction and Water Splitting. Small Methods 4, 1900586. 10.1002/smtd.201900586

[B22] Wang W. Ma M. Kong M. Yao Y. Wei N. (2017). Cobalt Carbonate Hydroxide Hydrate Nanowires Array: a Three‐dimensional Catalyst Electrode for Effective Water Oxidation. Micro Nano Lett. 12, 264–266. 10.1049/mnl.2016.0639

[B23] Wang Z.-J. Jin M.-X. Zhang L. Wang A.-J. Feng J.-J. (2021). Amorphous 3D Pomegranate-like NiCoFe Nanoassemblies Derived by Bi-component Cyanogel Reduction for Outstanding Oxygen Evolution Reaction. J. Energ. Chem. 53, 260–267. 10.1016/j.jechem.2020.05.026

[B24] Wu Z. P. Lu X. F. Zang S. Q. Lou X. W. (2020). Non‐Noble‐Metal‐Based Electrocatalysts toward the Oxygen Evolution Reaction. Adv. Funct. Mater. 30, 1910274. 10.1002/adfm.201910274

[B25] Xiao Y. Deng S. Li M. Zhou Q. Xu L. Zhang H. (2019). Immobilization of Fe-Doped Ni2P Particles within Biomass Agarose-Derived Porous N,P-Carbon Nanosheets for Efficient Bifunctional Oxygen Electrocatalysis. Front. Chem. 7, 523. 10.3389/fchem.2019.00523 31448255 PMC6691339

[B26] Xie Z. Zhang C. He X. Liang Y. Meng D. Wang J. (2019). Iron and Nickel Mixed Oxides Derived from NiIIFeII-PBA for Oxygen Evolution Electrocatalysis. Front. Chem. 7, 539. 10.3389/fchem.2019.00539 31428599 PMC6689985

[B27] Xu H. Cao J. Shan C. Wang B. Xi P. Liu W. (2018). MOF‐Derived Hollow CoS Decorated with CeO_X_ Nanoparticles for Boosting Oxygen Evolution Reaction Electrocatalysis. Angew. Chem. 130, 8790–8794. 10.1002/ange.201804673 29719107

[B28] Xu L. Jiang Q. Xiao Z. Li X. Huo J. Wang S. (2016). Plasma-Engraved Co_3_O_4_ Nanosheets with Oxygen Vacancies and High Surface Area for the Oxygen Evolution Reaction. Angew. Chem. 128, 5363–5367. 10.1002/ange.201600687 26990905

[B29] Yang L. Liu H. Shen H. Huang Y. Wang S. Zheng L. (2020). Physically Adsorbed Metal Ions in Porous Supports as Electrocatalysts for Oxygen Evolution Reaction. Adv. Funct. Mater. 30, 1909889. 10.1002/adfm.201909889

[B30] Zhang H. Wang J. Cheng Q. Saha P. Jiang H. (2020a). Highly Surface Electron-Deficient Co_9_S_8_ Nanoarrays for Enhanced Oxygen Evolution. Green. Energ. Environ. 5, 492–498. 10.1016/j.gee.2020.07.010

[B31] Zhang S. Huang B. Wang L. Zhang X. Zhu H. Zhu X. (2020b). Boosted Oxygen Evolution Reactivity via Atomic Iron Doping in Cobalt Carbonate Hydroxide Hydrate. ACS Appl. Mater. Inter. 12, 40220–40228. 10.1021/acsami.0c07260 32805817

[B32] Zhang Y. Xiao Q. Guo X. Zhang X. Xue Y. Jing L. (2015). A Novel Electrocatalyst for Oxygen Evolution Reaction Based on Rational Anchoring of Cobalt Carbonate Hydroxide Hydrate on Multiwall Carbon Nanotubes. J. Power Sourc. 278, 464–472. 10.1016/j.jpowsour.2014.12.092

[B33] Zhong H. Alberto Estudillo-Wong L. Gao Y. Feng Y. Alonso-Vante N. (2021). Oxygen Vacancies Engineering by Coordinating Oxygen-Buffering CeO_2_ with CoO Nanorods as Efficient Bifunctional Oxygen Electrode Electrocatalyst. J. Energ. Chem. 59, 615–625. 10.1016/j.jechem.2020.11.033

[B34] Zhu L. Wen Z. Mei W. Li Y. Ye Z. (2013). Porous CoO Nanostructure Arrays Converted from Rhombic Co(OH)F and Needle-like Co(CO_3_)_0.5_(OH)·0.11H_2_O and Their Electrochemical Properties. J. Phys. Chem. C 117, 20465–20473. 10.1021/jp406146b

