# Supplementary material for: Facile Synthesis of Carbon Cloth Supported Cobalt Carbonate Hydroxide Hydrate Nanoarrays for Highly Efficient Oxygen Evolution Reaction
Source: Front Chem. 2021 Aug 27;9:754357. doi: 10.3389/fchem.2021.754357 (PMC8429493; doi:10.3389/fchem.2021.754357)
Supplement: Supplementary file 1 [file DataSheet1.docx]

**Part I: Figures**


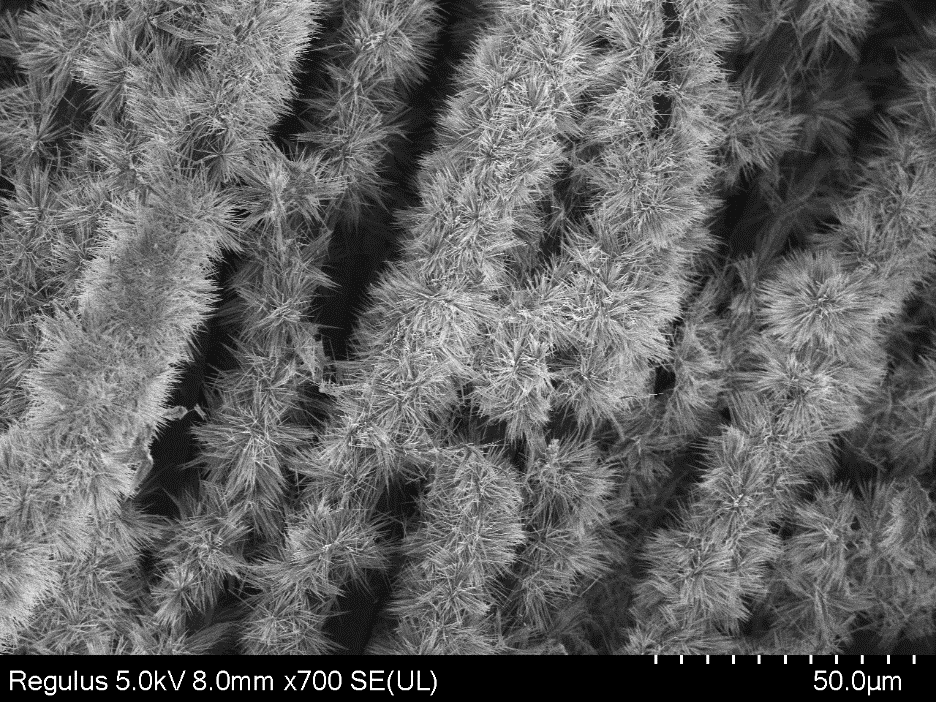


**Supplementary Figure 1.** SEM image of Co(CO_3_)_0.5_(OH)@CC nanoarrays.


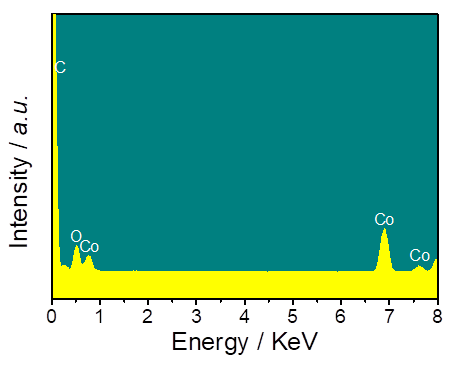


**Supplementary Figure 2.** EDX spectrum of Co(CO_3_)_0.5_(OH)@CC nanoarrays.

**
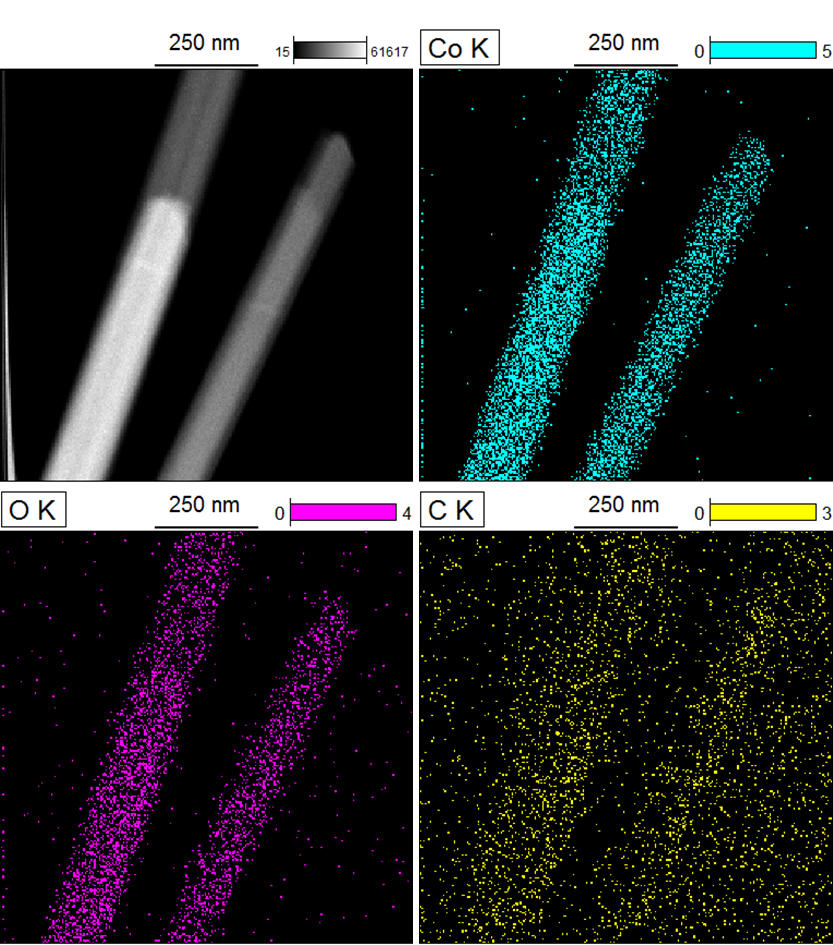
**

**Supplementary Figure 3.** Magnified STEM image and EDX element mappings of Co(CO_3_)_0.5_(OH) nanoneedles.

**
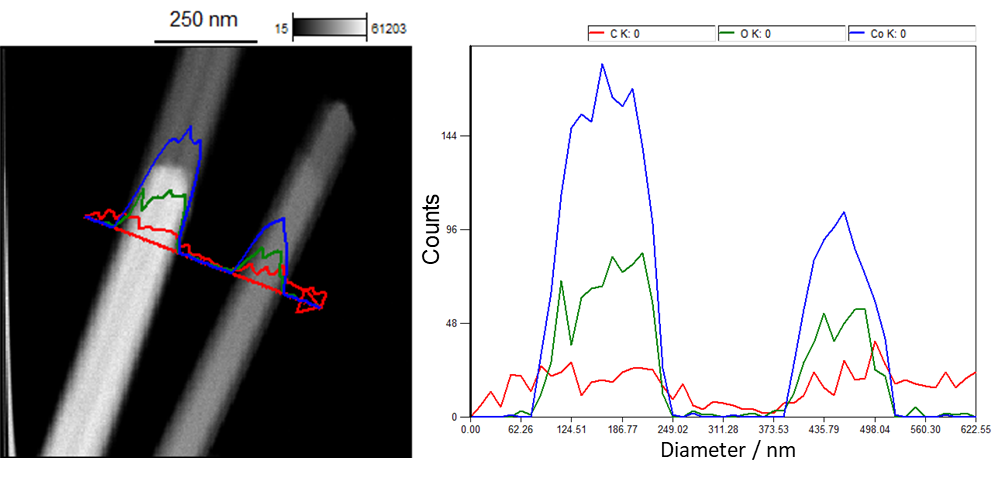
**

**Supplementary Figure 4.** Magnified STEM image and EDX line scanning profiles of Co(CO_3_)_0.5_(OH) nanoneedles.

**
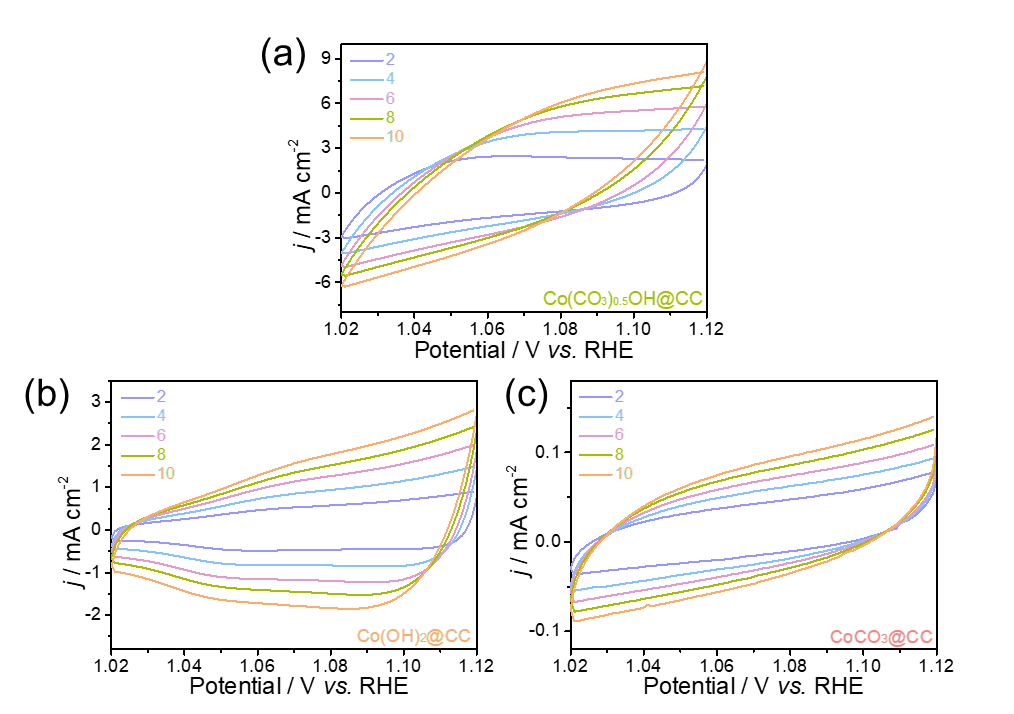
**

**Supplementary Figure 5.** CVs at different sweeping rates from 2 mV s^-1^ to 10 mV s^-1^ of (a) Co(CO_3_)_0.5_(OH)@CC, (b) Co(OH)_2_@CC and (c) CoCO_3_@CC.
